# Murine host response to *Neisseria gonorrhoeae* upper genital tract infection reveals a common transcriptional signature, plus distinct inflammatory responses that vary between reproductive cycle phases

**DOI:** 10.1186/s12864-018-5000-7

**Published:** 2018-08-22

**Authors:** Ian P. Francis, Epshita A. Islam, Adam C. Gower, Yazdani B. Shaik-Dasthagirisaheb, Scott D. Gray-Owen, Lee M. Wetzler

**Affiliations:** 1 Department of Medicine, Boston University School of Medicine, 715 Albany St. E-113, Boston, MA 02118 USA; 2 Department of Microbiology, Boston University School of Medicine, 72 E. Concord St., Room L504, Boston, MA 02118 USA; 3 Clinical and Translational Science Institute, Boston University School of Medicine, 715 Albany St. E-727, Boston, MA 02118 USA; 4 Department of Molecular Genetics, University of Toronto, Room 4383, Medical Sciences Building, 1 King’s College Circle, Toronto, ON M5S1A8 Canada

**Keywords:** *Neisseria gonorrhoeae*, Gonorrhea, Disease modeling, Murine reproductive cycle, Host immune response, Transcriptome, Microarray

## Abstract

**Background:**

The emergence of fully antimicrobial resistant *Neisseria gonorrhoeae* has led global public health agencies to identify a critical need for next generation anti-gonococcal pharmaceuticals. The development and success of these compounds will rely upon valid pre-clinical models of gonorrhoeae infection. We recently developed and reported the first model of upper genital tract gonococcal infection. During initial characterization, we observed significant reproductive cycle-based variation in infection outcome. When uterine infection occurred in the diestrus phase, there was significantly greater pathology than during estrus phase. The aim of this study was to evaluate transcriptional profiles of infected uterine tissue from mice in either estrus or diestrus phase in order to elucidate possible mechanisms for these differences.

**Results:**

Genes and biological pathways with phase-independent induction during infection showed a chemokine dominant cytokine response to *Neisseria gonorrhoeae*. Despite general induction being phase-independent, this common anti-gonococcal response demonstrated greater induction during diestrus phase infection. Greater activity of granulocyte adhesion and diapedesis regulators during diestrus infection, particularly in chemokines and diapedesis regulators, was also shown. In addition to a greater induction of the common anti-gonococcal response, Gene Set Enrichment Analysis identified a diestrus-specific induction of type-1 interferon signaling pathways.

**Conclusions:**

This transcriptional analysis of murine uterine gonococcal infection during distinct points in the natural reproductive cycle provided evidence for a common anti-gonococcal response characterized by significant induction of granulocyte chemokine expression and high proinflammatory mediators. The basic biology of this host response to *N. gonorrhoeae* in estrus and diestrus is similar at the pathway level but varies drastically in magnitude. Overlaying this, we observed type-1 interferon induction specifically in diestrus infection where greater pathology is observed. This supports recent work suggesting this pathway has a significant, possibly host-detrimental, function in gonococcal infection. Together these findings lay the groundwork for further examination of the role of interferons in gonococcal infection. Additionally, this work enables the implementation of the diestrus uterine infection model using the newly characterized host response as a marker of pathology and its prevention as a correlate of candidate vaccine efficacy and ability to protect against the devastating consequences of *N. gonorrhoeae*-associated sequelae.

**Electronic supplementary material:**

The online version of this article (10.1186/s12864-018-5000-7) contains supplementary material, which is available to authorized users.

## Background

Gonorrhea, caused by the human-restricted Gram-negative bacterium *Neisseria gonorrhoeae* (also known as the gonococcus), is a rapidly worsening public health threat. While uncomplicated gonorrhea typically manifests as urethritis in men and cervicitis in women, a significant subset of women develop more serious sequelae. Approximately 15% of all infected untreated women develop pelvic inflammatory disease (PID) [1]. PID is a serious inflammatory condition that can cause fibrosis and scarring of the upper genital tract (UGT), resulting in long term complications including chronic pelvic pain, infertility, increased risk of ectopic pregnancy and endometriosis. Additionally, active gonorrhea is known to increase HIV replication and transmission rates [2, 3]. This is a serious consideration in the fight to end HIV transmission since *N. gonorrhoeae* has such a large and widespread disease burden. In 2016, over 400,000 new cases of gonococcal infection were reported to the Centers for Disease Control and Prevention (CDC), while the World Health Organization (WHO) places global yearly incidence at 106.1 million [4, 5]. Alarmingly, these figures may under-estimate the actual disease burden by up to two-thirds [6].

While major complications and uncontrolled transmission have been largely prevented during the antibiotic era, there is significant evidence to suggest that this era is coming to an end [7, 8]. Beginning with penicillin, *N. gonorrhoeae* has repeatedly demonstrated an ability to develop resistance, through a variety of mechanisms, to whatever principal therapeutic is in use [9–17]. Once again, this pattern is repeating with global clinical isolates demonstrating consistently increasing minimum inhibitory concentrations (MICs) to ceftriaxone/azithromycin combination therapy approaching the threshold of resistance [7, 17]. This situation is even more alarming than previous examples of acquired resistance because this therapy is the final treatment identified as efficacious by the CDC [18]. This threat of fully drug-resistant *N. gonorrhoeae* is not hypothetical; strains resistant to cefixime, ceftriaxone, and azithromycin have already been reported throughout the world with increasing frequency over the past 15 years [19–21]. In response to this looming threat, both the CDC and the WHO have identified *N. gonorrhoeae* as a critical-level public health threat requiring immediate development of novel therapeutics [22, 23]. Unfortunately, new anti-gonococcal strategies, either antimicrobials or vaccines, are not yet evident on the horizon.

As *N. gonorrhoeae* is highly adapted to life in humans [24–26], modeling this infection in in vivo lab models is extremely difficult. Early studies of infection and disease utilized human volunteers or chimpanzees; however, modern ethical standards, prohibitive cost, and experimental limitations have rendered them non-viable for application in current research [24, 27–29]. Since its introduction in 1999, the female mouse lower-genital tract vaginal colonization model has been the primary tool for in vivo experimentation with GC [30, 31]. Notably, bacteria are rapidly cleared during the diestrus phase of the murine reproductive cycle, which means the model mouse must be arrested in the estrus phase, and GC persistence is additionally facilitated by suppression of the natural vaginal microbiome using antibiotics [30]. This estrus infection model displays a significant, albeit mild, induction of pro-inflammatory cytokines and influx of neutrophils following infection [30–32]. This well-established model is, therefore, reminiscent of the asymptomatic colonization in women rather than active lower genital tract infection or PID.

While asymptomatic colonization may be the short-term outcome of most infections in women, the threat of fully antibiotic resistant GC has shifted the focus of therapeutic/vaccine development to the more serious endpoints associated with chronic infection, like PID. Although prevention of colonization and transmission are the clear goals of any novel therapeutic agent or vaccine, prevention of these serious endpoints are of primary focus in the context of fully drug resistant bacteria. With only the murine estrus vaginal colonization model available, drug and vaccine discovery efforts have been unable to directly evaluate the efficacy of their products in preventing the pathology associated with gonorrhoeae or PID. To overcome this barrier, we have recently described a model whereby transcervical infection of female mice during the diestrus phase leads to rapid GC penetration into the tissues and very high levels of inflammatory cytokines, and a correspondingly profound recruitment of neutrophils into the infected tissue reminiscent of that seen during human PID [33]. Remarkably, when mice are in estrus phase, which supports lower genital tract colonization, they show little sign of inflammation and no infection-associated pathology following transcervical infection. The fact that infection with the same bacterium can induce such strikingly different phenotypes in systems that differ only in their natural reproductive hormone cycle phase raises fascinating questions regarding the driving forces behind these processes. This study aims to use comparative genome-wide transcriptional profiling of host gene expression during upper genital tract infection with GC during the estrus and diestrus phases to understand these markedly different outcomes.

## Results

### Experimental design

To reveal any reproductive cycle phase-dependent transcriptional differences in host response to GC upper genital tract infection, we compared transcriptional profiles from uterine tissue collected from mice in 4 distinct experimental groups; transcervically-administered PBS mice during diestrus phase (*N* = 4), transcervically-administered GC infected mice during diestrus phase (N = 4), transcervically-administered PBS treated mice during estrus phase (*N* = 3), and transcervically-administered GC infected mice during estrus phase (N = 4). Following protocols used in previous work in our lab, all tissue was collected 6 h post-treatment to capture the acute response that is characteristic of this model.

To best examine the impact of our two variables (reproductive-cycle phase and infection state) independently and together, we modeled gene expression as a linear function of reproductive-cycle phase, infection state, and the interaction between phase and infection state (phase:infection). For each model, moderated *t* tests were performed on the corresponding coefficient of the linear model to obtain a *t* statistic and *p* value for each gene. In order to account for multiple comparison testing error, Benjamini-Hochberg false discovery rate (FDR) correction was then applied to obtain corrected *p* values (*q* values) after removing genes that were not expressed above the median value of at least one array. Finally, only results achieving the standard cut off of an FDR *q* less than 0.25 were included subsequent analyses or conclusions. This analysis identifies genes whose expression is significantly impacted by one of our variables, after correcting for the effect of the other; or in the case of phase:infection interaction, the *t* statistic generated measured the significance of a combined effect of the variables on a genes expression.

### Linear modeling allows for accurate directed comparisons of gene expression in a complex system

To evaluate the ability of our analytical approach to identify differential patterns in biological processes in this multi-variable transcriptional data set, we examined those genes identified as having expression significantly associated with reproductive cycle phase in all samples regardless of infection status. The physiological state of uterine tissue is extremely different at distinct points in the reproductive cycle. This was reflected by the large number of genes that were significantly associated with reproductive cycle phase (11,310 genes at FDR *q* < 0.25; Additional file 1). To evaluate differences in biological processes between reproductive cycle phases, we used the phase *t* statistic to perform pre-ranked Gene Set Enrichment Analysis (GSEA) [34, 35]. GSEA identified 732 gene sets that were significantly (FDR *q* < 0.25) coordinately upregulated in diestrus phase compared to estrus phase after correcting for the effect of GC infection (Additional file 2). The ten gene sets most significantly associated with reproductive cycle phase (Table 1) are representative of these 732 gene sets which are primarily cell cycle related processes. This reflects the fact that the uterus undergoes profound dynamic changes in its cellular and structural state throughout the reproductive cycle. Similar reproductive phase dependent transcriptional differences in biological processes have been previously described in human uterine tissue [36]. Knowing that our system allows for accurate and directed exploration of the transcriptional data we turned our attention to examining transcriptional changes in GC infection.

**Table 1:** Phase dependent genes are significantly associated with cell cycle processes

Gene set source and type	Gene Set Name	Normalized Enrichment Score (NES)	Nominal *p* value	FDR q value
Reactome pathway	REACTOME_MITOTIC_M_M_G1_PHASES	2.86	0.0000	0.0000
Reactome pathway	REACTOME_CELL_CYCLE_MITOTIC	2.82	0.0000	0.0000
Reactome pathway	REACTOME_DNA_REPLICATION	2.80	0.0000	0.0000
Reactome pathway	REACTOME_CELL_CYCLE	2.76	0.0000	0.0000
KEGG pathway	KEGG_CELL_CYCLE	2.67	0.0000	0.0000
Reactome pathway	REACTOME_MITOTIC_PROMETAPHASE	2.66	0.0000	0.0000
Reactome pathway	REACTOME_G2_M_CHECKPOINTS	2.65	0.0000	0.0000
Reactome pathway	REACTOME_ACTIVATION_OF_ATR_IN_RESPONSE_TO_REPLICATION_STRESS	2.60	0.0000	0.0000
GO Biological Process	DNA_REPLICATION	2.57	0.0000	0.0000

The differential activity of biologic systems in those genes identified as having significant reproductive-cycle-phase-effected expression was evaluated by GSEA. The biological processes represented in the 732 gene sets identified by GSEA as containing genes whose expression demonstrated significant phase effect are reflected in these 10 most significantly associated gene sets. Gene sets are ranked in descending order by Normalized Enrichment score and are labeled according to the MSigDB sub-collection to which they belong. The full table of identified gene sets can be found in Additional file 2

### *Neisseria gonorrhoeae* infection induces expression of immune response genes regardless of hormone cycle phase

**Fig. 1:**
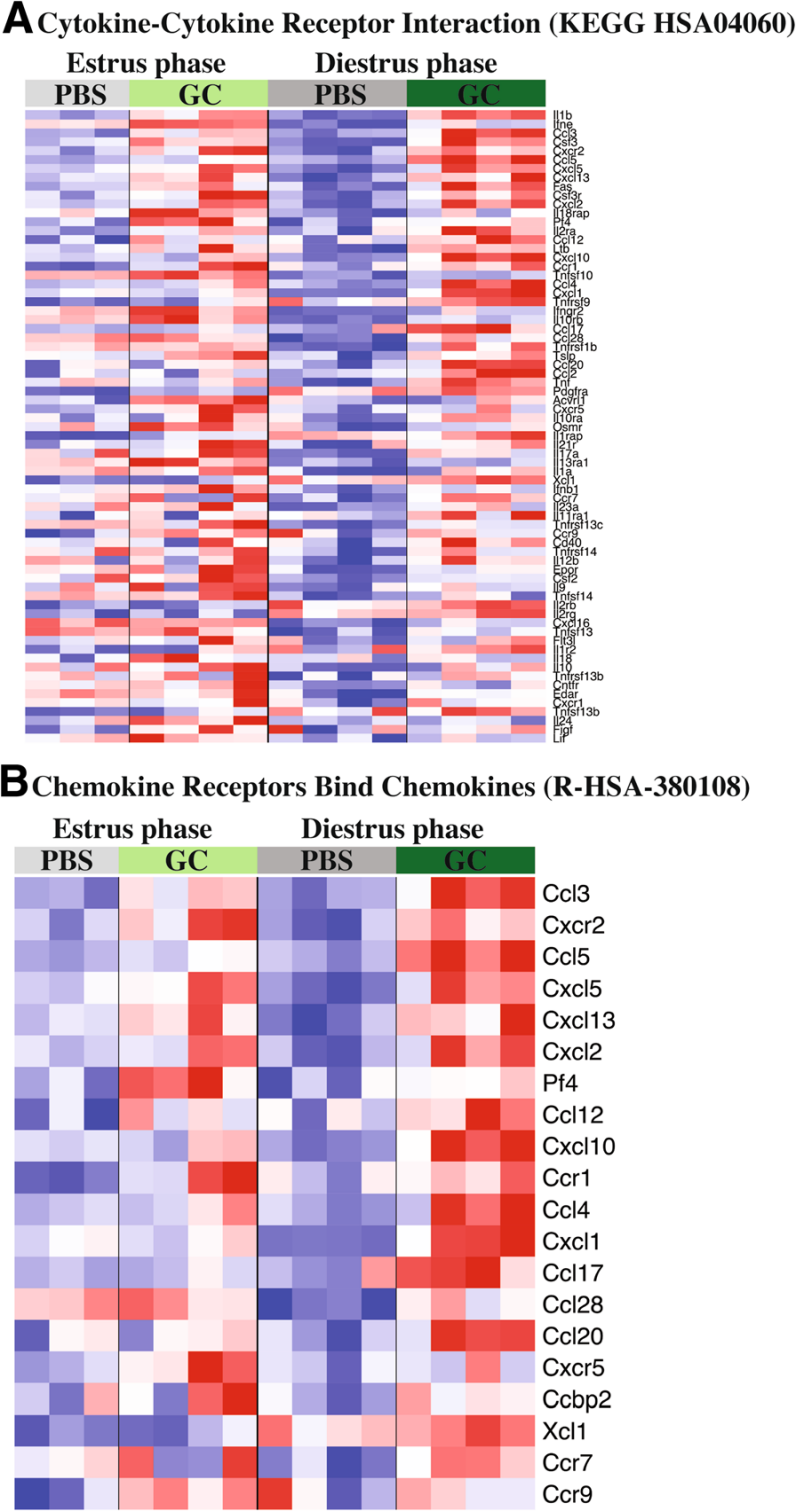
Leading edge genes from top phase-effect gene sets show phase-independent induction but phase-dependent induction magnitude. The infection dependent host response was evaluated by microarray analysis of mRNA extracted from infected uterine tissue collected 6 h after initial treatment with 10^7^ gonococci or PBS. Expression levels of leading edge genes from for top gene sets identified by infection-effect GSEA are presented; “Cytokine-Cytokine Receptor Interaction” (KEGG HSA04060) (**a**) and “Chemokine Receptors Bind Chemokines” (R-HSA-380108) (**b**). For each gene, expression values (log2(expression)) are normalized to a mean of zero and standard deviation of one (z-normalized) for visual representation so that red and blue indicate z-scores of ≥2 or ≤ − 2, respectively, and white indicates a z-score of 0 (row-wise mean). Genes are presented in descending significance of infection effect *t* statistic (top to bottom). (*N* = 4 per condition except *N* = 3 for PBS Estrus)

Using the previously described linear modeling approach, this time modeling expression as a function of infection state, we identified a large population of genes whose expression was significantly associated with infection state after correcting for reproductive cycle effects (2244 genes with FDR *q* < 0.25). The biological context of those 2244 genes was provided by pre-ranked GSEA performed using the infection *t* statistics, which identified 449 gene sets that showed significant (FDR *q* < 0.25) coordinate expressional regulation with respect to infection (Additional file 3). Those gene sets that demonstrated the most significant positive coordination of expression (or upregulation in infected compared to uninfected tissues) were almost exclusively related to the host immune response, including the gene sets “chemokine receptors bind chemokines” (Reactome; R-HSA-380108) and “cytokine cytokine-receptor interaction” (KEGG; HSA04060). The genes from each gene set that most contributed to the significance of the set’s infection effect, referred to as the leading-edge genes, exhibit an expression pattern indicative of significant infection effect (Fig. 1). The cytokine gene sets demonstrate clear phase-independent induction with expression in infected tissues being appreciably higher than their uninfected phase-matched controls. Several of the cytokines and chemokines found at the leading edge of these sets are suggestive of gonorrhea’s characteristic recruitment of leukocytes to infected tissues. These genes include the neutrophil chemokines *Cxcl5*, *Ccl4* and *Cxcl1*, as well as the T cell chemokines *Ccl5*, *Cxcl10* and *Ccl17*. Gonorrhea’s strong inflammatory reaction was also reflected in this phase-independent anti-GC response with classic proinflammatory mediators like *Il1a* and *Il1b*, *Ltb* (Lymphotoxin Beta), and *Tnf* (TNF-α) found among the leading-edge genes. The presence of immune response gene sets, driven by proinflammatory cytokine and chemokine expression induction, in the infection effect GSEA suggests an anti-gonococcal response common to all reproductive phases characterized by local inflammation and immune cell invasion. Despite the apparent universality of this response to GC infection, our previous descriptions of profound phenotypic differences, in these same pathways, between infection during estrus and diestrus phases suggests a more complex process at work.

These differences in infection phenotype may be at least partially due to differential magnitudes of activation of this common anti-gonococcal response. Evidence of this can be found in the same genes highlighted as the hallmarks of the general anti-GC response, as they display unequal induction by infection. This is particularly striking with the neutrophil chemokines. For example, *Cxcl5*, while upregulated in all infected tissues, expression in diestrus tissue is lower at baseline and greater in infection as compared to estrus (Fig. 1b). This greater magnitude expression in diestrus is found generally in both the chemokine and cytokine gene sets (Fig. 1). These observations indicate that unequal activation of a common anti-gonococcal host response, characterized by inflammatory and cell recruitment processes, at least partially is responsible for phenotypic differences between infected estrus and diestrus tissue.

### Genes exhibiting significant reproductive cycle-dependent infection responses separate into distinct expression patterns

**Fig. 2:**
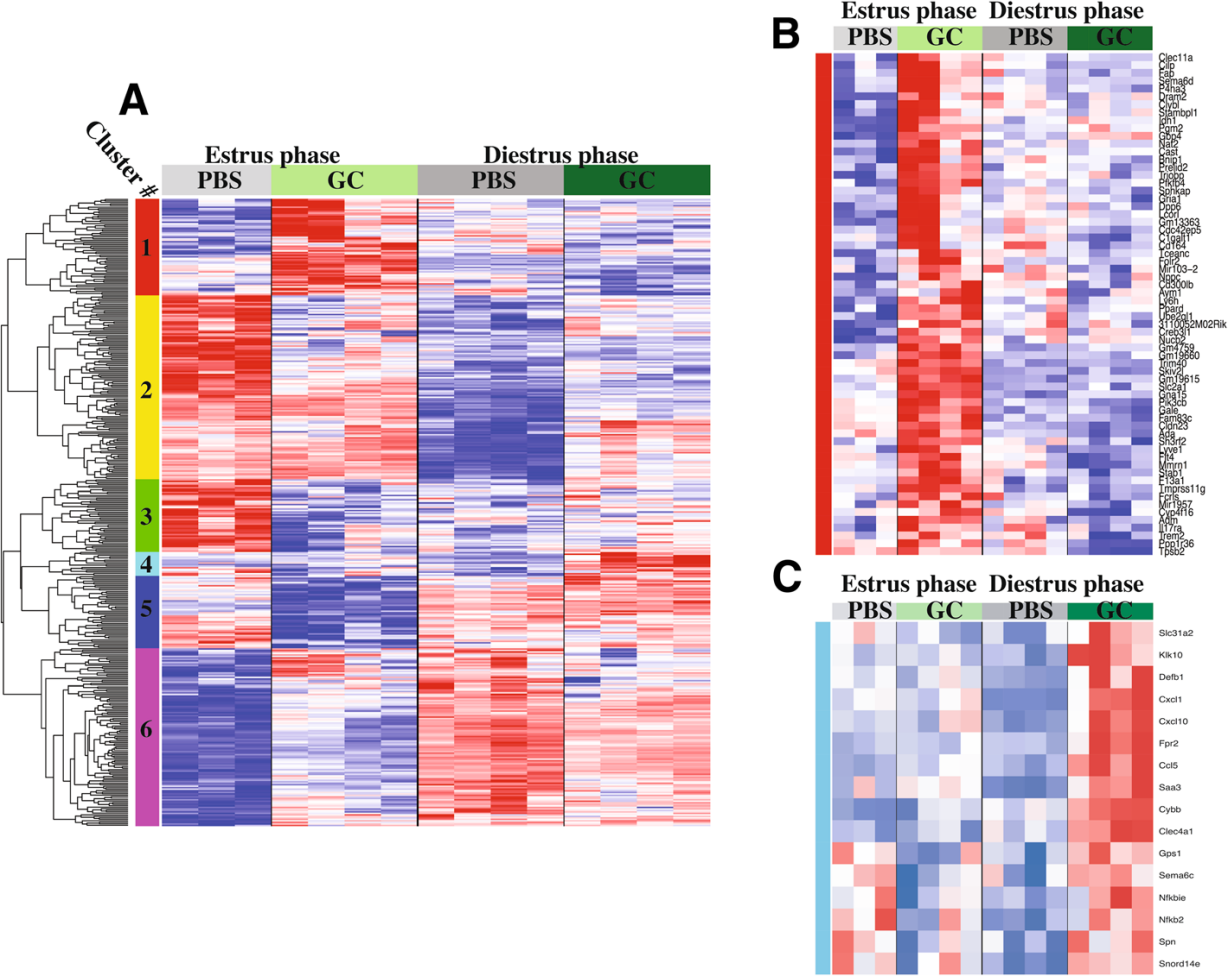
Genes with significant phase:infection interaction effect cluster into distinct expression patterns. Reproductive phase dependent elements of the anti-GC host response were evaluated through identification of phase:infection effected genes within microarray analysis of mRNA extracted from infected uterine tissue collected 6 h after initial treatment with 10^7^ gonococci or PBS. Expression levels of (**a**) All 416 genes with phase:infection FDR *q* < 0.25 were clustered based on their relative expression pattern across all samples. Clusters are indicated by colored sidebar and number. Rows represent genes, with log2(expression) values z-normalized (to a mean of zero and a standard deviation of one) across all samples. Colors are scaled so that red and blue indicate z-scores of ≥2 or ≤ − 2, respectively, and white indicates a z-score of 0 (row-wise mean). (**b**) Enlarged view of Cluster 1, comprised of genes induced specifically in estrus phase. (**c**) Enlarged view of Cluster 4, comprised of genes induced specifically in diestrus phase. N = 4 for all conditions except N = 3 for PBS Estrus

To determine whether there were also reproductive-phase-dependent transcriptional differences in the host response to transcervical infection, we applied the same analytical approach as employed above for phase and infection effect. For this analysis however, the modeled linear function was expression as a function of the interaction between reproductive-cycle phase and infection state (phase:infection). Applying moderated *t* tests on the resulting coefficient of the linear model we were able to identify genes that had different expressional changes between the two reproductive-cycle phases, in response to infection. This analysis identified 416 genes (FDR *q* < 0.25) subject to significant phase:infection effect, which clustered into 6 distinct patterns of expression (Fig. 2a).

Genes with increased expression only in estrus-phase infection are found in cluster 1 and represent diverse biological functions (Fig. 2b). Of the 64 genes in cluster 1, only a few are potentially immunologically significant and these do not strongly suggest specific estrus-infection immune processes. The low affinity IL17 receptor, encoded for by *IL17ra*, is found in cluster 1. Since this cytokine has demonstrated a wide variety of functions and this specific receptor itself has been tied to cutaneous homeostasis, mucosal immune responses, and potentiation of antibody driven autoimmunity, it is difficult to interpret its presence in this cluster without additional members of any of those biological pathways [37–41]. Similar ambiguity of function surrounds the other immune genes found in cluster 1. The activating receptor encoded for by *Cd300lb* can be found both in myeloid cell membranes or secreted, serving two distinct functions [42, 43]. While the lack of corroborating genes in cluster 1 obscures the role of this gene in the less inflammatory phenotype of estrus GC colonization, the literature may explain the increased *Cd300lb* transcript levels. Previous work has described the increased expression and secretion of the receptor by neutrophils in response to LPS [43]. It is possible the same effect is induced in the particularly large neutrophil population present at baseline in the murine uterus during estrus. Perhaps the most intriguing immune gene found in cluster 1 is *Trem2*. This gene encodes for an anti-inflammatory receptor expressed on innate immune cells that bind to and respond specifically to LPS [44]. Like with the previously discussed genes, it is hard to comment on any broader biological impact of the increased expression of *Trem2* without coincident increases in known co-mediators of a given pathway, however the strong anti-inflammatory function of this receptor suggest that it could contribute to the dampened inflammatory response observed in GC infection during estrus [33].

The remaining genes in the cluster are associated with either non-immunologically relevant pathways or broad non-specific functions with minor (if any) immunological implications. The latter is best exemplified by *Pik3cb*, which encodes an isoform of a regulatory protein in the phosphatidylinositol signaling system, an expansive system with some components active in immune processes. Other processes represented in cluster 1 include metabolism (e.g. Pfkfb4) and extracellular structure (e.g. P4ha3), which reflect the baseline biologic functions in the estrus uterine tissue (buildup and maintenance of the thick uterine lining) [36].

Cluster 4 encompasses transcripts upregulated only during diestrus-phase infection. In contrast to the estrus-restricted responses of cluster 1, this cluster is comprised almost entirely of immunologically active genes (Fig. 2c). These include the previously discussed chemokines Cxcl1, Cxcl10, and Ccl5; the formyl peptide-specific chemoattractant receptor Fpr2, which has an identified role in host response to some bacterial infections [45]; the endogenous antimicrobials beta-defensin 1 (Defb1) and reactive-oxygen-species (ROS)-producing enzyme NADPH Oxidase (NOX2; Cybb); and major regulators of general immune activation including a component of the classic pro-inflammatory transcription factor, Nuclear Factor kappa-B (Nfkb2). These findings lend transcriptomic context for the stark phenotypic differences observed in mice transcervically infected with GC during the diestrus and estrus phases of the reproductive cycle. The remarkable absence of immune response genes induced exclusively in estrus infection suggests a lack of a unique anti-bacterial host response beyond the previously discussed common anti-gonococcal response. This stands in stark contrast to the diestrus infection specific induction of a cohesive set of genes that clearly indicate induction of specific host response pathways including immune cell effector function and anti-bacterial action, in addition to the cell recruitment and inflammation of the common anti-gonococcal response.

### Infection in diestrus phase induces members of immunologically relevant gene sets

In order to fully evaluate differences in biological processes associated with GC infection in different reproductive-cycle phases, we once again performed a pre-ranked GSEA, this time using the phase:infection interaction *t* statistic [34, 35]. This GSEA identified 70 gene sets with significant (FDR *q* < 0.25) coordinate expression of genes displaying a pattern of greater upregulation (or less downregulation) in diestrus phase infection than in estrus phase infection (Table 2 and Additional file 4). Notably, the significant gene sets included not only those suggestive of the inflammation and cell recruitment focused common anti-gonococcal response, but additional sets suggesting greater type I interferon signaling (Table 2 set: “Interferon alpha beta signaling”), pattern recognition receptor (PRRs) activity (Table 2 sets: “TLR signaling pathway”, “NLR signaling pathway”, “Detection of a stimulus”), and immune cell activation/function (Table 2 sets: “CD40 pathway”, “NFKB pathway”, “Myeloid cell differentiation”, “Leukocyte differentiation”, “Immune Effector Process”) in diestrus infection. The top sets in the GSEA indicated particular activation of interferon and chemokine activity.

**Table 2:** Gene sets significantly associated with positive phase:infection *t* statistics

Gene set source and type	Gene Set Name	Normalized Enrichment Score (NES)	Nominal *p* value	FDR q value
Reactome pathway	REACTOME_INTERFERON_ALPHA_BETA_SIGNALING	2.50	0.000	0.000
GO Molecular Function	GO CHEMOKINE_ACTIVITY	2.34	0.000	0.000
GO Molecular Function	GO CHEMOKINE_RECEPTOR_BINDING	2.19	0.000	0.001
GO Molecular Function	GO CALMODULIN_BINDING	1.99	0.000	0.045
GO Biological Process	GO RESPONSE_TO_OTHER_ORGANISM	1.99	0.000	0.037
GO Biological Process	GO MYELOID_CELL_DIFFERENTIATION	1.97	0.000	0.040
KEGG pathway	KEGG_OLFACTORY_TRANSDUCTION	1.95	0.000	0.043
GO Molecular Function	GO G_PROTEIN_COUPLED_RECEPTOR_BINDING	1.95	0.000	0.038
BioCarta pathway	BIOCARTA_NFKB_PATHWAY	1.95	0.000	0.036
GO Molecular Function	GO ANION_TRANSMEMBRANE_TRANSPORTER_ACTIVITY	1.93	0.000	0.040
BioCarta pathway	BIOCARTA_TNFR2_PATHWAY	1.93	0.000	0.038
KEGG pathway	KEGG_TOLL_LIKE_RECEPTOR_SIGNALING_PATHWAY	1.91	0.002	0.047
Reactome pathway	REACTOME_CGMP_EFFECTS	1.90	0.002	0.049
GO Biological Process	RESPONSE_TO_VIRUS	1.87	0.002	0.064
KEGG pathway	KEGG_CYTOSOLIC_DNA_SENSING_PATHWAY	1.86	0.000	0.071
KEGG pathway	KEGG_TYPE_I_DIABETES_MELLITUS	1.85	0.002	0.076
KEGG pathway	KEGG_NOD_LIKE_RECEPTOR_SIGNALING_PATHWAY	1.84	0.002	0.075
Reactome pathway	REACTOME_OLFACTORY_SIGNALING_PATHWAY	1.84	0.000	0.072
Reactome pathway	REACTOME_TRAF6_MEDIATED_IRF7_ACTIVATION	1.82	0.004	0.087
GO Biological Process	GO DETECTION_OF_STIMULUS	1.81	0.000	0.092
GO Molecular Function	GO CYTOKINE_ACTIVITY	1.81	0.000	0.095
TF motif	GOGTTRYCATRR_UNKNOWN	1.80	0.000	0.097
BioCarta pathway	BIOCARTA_TALL1_PATHWAY	1.79	0.009	0.098
Reactome pathway	REACTOME_INTERFERON_SIGNALING	1.79	0.000	0.097
KEGG pathway	KEGG_RIG_I_LIKE_RECEPTOR_SIGNALING_PATHWAY	1.79	0.000	0.095
Reactome pathway	REACTOME_NUCLEOTIDE_BINDING_DOMAIN_LEUCINE_RICH_REPEAT_CONTAINING_RECEPTOR_NLR_SIGNALING_PATHWAYS	1.78	0.004	0.097
TF motif	STTTCRNTTT_V$IRF_Q6	1.78	0.000	0.095
Reactome pathway	REACTOME_RIG_I_MDA5_MEDIATED_INDUCTION_OF_IFN_ALPHA_BETA_PATHWAYS	1.78	0.000	0.093
GO Biological Process	DEFENSE_RESPONSE	1.78	0.000	0.090
Reactome pathway	REACTOME_DEGRADATION_OF_THE_EXTRACELLULAR_MATRIX	1.77	0.003	0.094
Reactome pathway	REACTOME_NITRIC_OXIDE_STIMULATES_GUANYLATE_CYCLASE	1.77	0.007	0.096
Reactome pathway	REACTOME_HS_GAG_BIOSYNTHESIS	1.76	0.007	0.098
TF motif	GGGNNTTTCC_V$NFKB_Q6_01	1.73	0.000	0.132
GO Biological Process	INFLAMMATORY_RESPONSE	1.72	0.000	0.141
BioCarta pathway	BIOCARTA_IL1R_PATHWAY	1.72	0.009	0.138
BioCarta pathway	BIOCARTA_CD40_PATHWAY	1.72	0.007	0.138
Reactome pathway	REACTOME_INTERFERON_GAMMA_SIGNALING	1.71	0.003	0.135
KEGG pathway	KEGG_ARRHYTHMOGENIC_RIGHT_VENTRICULAR_CARDIOMYOPATHY_ARVC	1.71	0.000	0.143
GO Cellular Component	NUCLEAR_CHROMOSOME	1.70	0.007	0.151
GO Biological Process	LEUKOCYTE_DIFFERENTIATION	1.70	0.002	0.147
GO Biological Process	RESPONSE_TO_BIOTIC_STIMULUS	1.69	0.000	0.153
GO Biological Process	IMMUNE_SYSTEM_DEVELOPMENT	1.69	0.004	0.154
GO Biological Process	IMMUNE_EFFECTOR_PROCESS	1.68	0.009	0.157
GO Biological Process	CHROMATIN_MODIFICATION	1.68	0.004	0.160
BioCarta pathway	BIOCARTA_TID_PATHWAY	1.68	0.013	0.159
GO Molecular Function	CHLORIDE_CHANNEL_ACTIVITY	1.66	0.007	0.175
GO Molecular Function	ANION_CHANNEL_ACTIVITY	1.66	0.011	0.178
Reactome pathway	REACTOME_PRE_NOTCH_TRANSCRIPTION_AND_TRANSLATION	1.66	0.016	0.177
GO Biological Process	AMINO_SUGAR_METABOLIC_PROCESS	1.65	0.007	0.182
GO Biological Process	REGULATION_OF_MYELOID_CELL_DIFFERENTIATION	1.64	0.010	0.204
KEGG pathway	KEGG_DILATED_CARDIOMYOPATHY	1.63	0.000	0.202
TF motif	V$NFKAPPAB_01	1.63	0.000	0.202
GO Biological Process	HISTONE_MODIFICATION	1.63	0.020	0.201
Reactome pathway	REACTOME_DNA_STRAND_ELONGATION	1.63	0.020	0.203
GO Biological Process	HEMOPOIETIC_OR_LYMPHOID_ORGAN_DEVELOPMENT	1.63	0.000	0.203
GO Biological Process	HEMOPOIESIS	1.62	0.000	0.204
GO Biological Process	COVALENT_CHROMATIN_MODIFICATION	1.62	0.022	0.201
BioCarta pathway	BIOCARTA_NTHI_PATHWAY	1.62	0.021	0.209
Reactome pathway	REACTOME_TRAF6_MEDIATED_NFKB_ACTIVATION	1.61	0.020	0.212
GO Biological Process	ESTABLISHMENT_AND_OR_MAINTENANCE_OF_CHROMATIN_ARCHITECTURE	1.61	0.007	0.213
GO Biological Process	DETECTION_OF_EXTERNAL_STIMULUS	1.61	0.025	0.210
GO Cellular Component	EXTRACELLULAR_SPACE	1.60	0.000	0.229
GO Biological Process	RESPONSE_TO_BACTERIUM	1.59	0.033	0.234
Reactome pathway	REACTOME_HEPARAN_SULFATE_HEPARIN_HS_GAG_METABOLISM	1.59	0.014	0.233
GO Molecular Function	SYMPORTER_ACTIVITY	1.59	0.020	0.238
TF motif	V$CREL_01	1.58	0.000	0.239
GO Biological Process	RESPONSE_TO_UV	1.58	0.025	0.237
BioCarta pathway	BIOCARTA_STRESS_PATHWAY	1.58	0.019	0.234
KEGG pathway	KEGG_ALLOGRAFT_REJECTION	1.58	0.026	0.246
GO Biological Process	MORPHOGENESIS_OF_AN_EPITHELIUM	1.58	0.029	0.242

The differential activity of biologic systems within the phase:infection interaction effected genes was evaluated by GSEA. Seventy gene sets were identified as significantly (FDR *q* < 0.25) coordinately up-regulated to a greater degree (or down-regulated to a lesser degree) during infection in diestrus phase than in estrus phase. Gene sets are ranked in descending order by Normalized Enrichment score, and are labeled according to the MSigDB sub-collection to which they belong

**Fig. 3:**
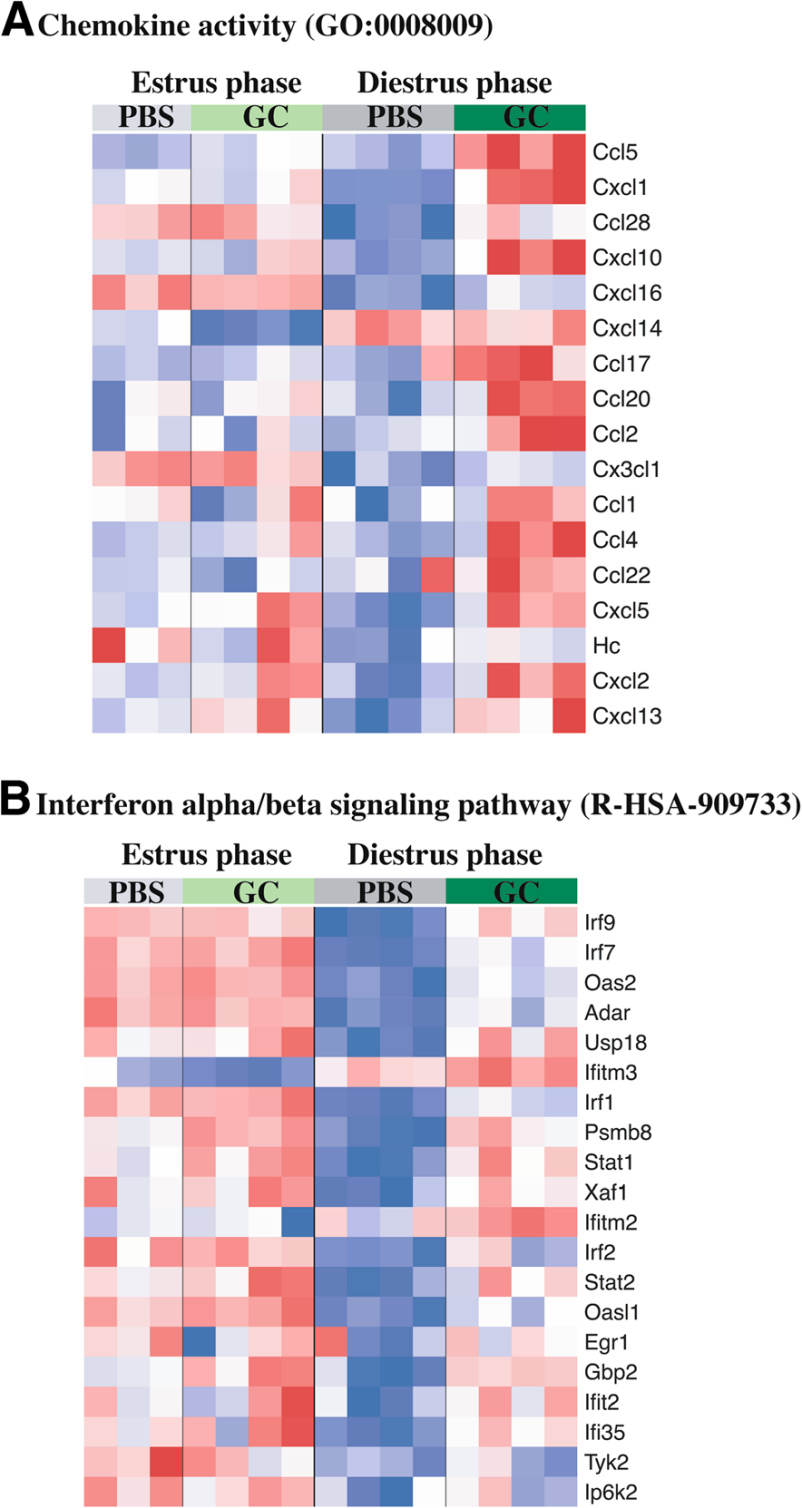
Leading edge genes from top phase:infection interaction-effect gene sets. Transcript levels of leading edge genes, from GSEA identified top phase:infection interaction effect gene sets (**a.** Chemokine activity gene set [GO:0008009], **b.** Interferon alpha/beta signaling pathway [R-HSA-909733]), as measured 6 h after infection, are displayed by heatmap. Rows represent genes, with log2(expression) values z-normalized (to a mean of zero and a standard deviation of one) across all samples. Colors are scaled so that red and blue indicate z-scores of ≥2 or ≤ − 2, respectively, and white indicates a z-score of 0 (row-wise mean). Rows are arranged in descending order from top to bottom by phase:infection *t* statistic. N = 4 per condition except PBS estrus N = 3

To better evaluate gene expression patterns in top GSEA sets, we examined their leading-edge genes (Fig. 3). The leading-edge genes of the chemokine activity gene set (GO term GO:0008009) (Fig. 3a) repeat the same pattern that has been seen throughout the analysis: induction by infection over low levels of expression in uninfected tissue that is much greater in magnitude in diestrus phase. In fact, several of the cytokines seen here are the same neutrophil chemokines (Cxcl5, Ccl4, Cxcl1) and T-cell chemokines (Cccl5, Cxcl10, Ccl17) that were highlighted in the infection effect analysis (Fig. 1b). This inclusion of similar gene sets containing the same genes reinforces the greater induction of the common anti-gonococcal response in diestrus. The chemokine with one of the greatest differences in estrus and diestrus infection dependent induction was *Ccl20*. This lymphocyte chemokine is active in the mucosal adaptive immune response in the gastrointestinal tract, particularly in response to bacterial infection [46, 47] [48, 49]. While we could not find any description of CCL20 induction by GC specifically, there is evidence that production of this chemokine can be induced by bacterial products [50] and is suppressed by estrogen [51], which together would explain its particularly potent induction in diestrus infection.

In contrast to the cytokine gene set, most of the leading-edge genes in the interferon alpha/beta signaling gene set (Reactome pathway R-HSA-909733) (Fig. 3b) displayed very little infection-dependent induction in estrus, but robust diestrus-phase induction by infection. The leading-edge genes suggest a fully mature interferon response active in diestrus infected tissue, with induction of positive regulators of type one interferon expression (*Irf1*, *Irf7*), signaling (*Irf9*, *Stat1* and *Stat2*), effector function (*Ifitm2*, *Ifitm3*, *Gbp2*, and *Ifi35*), and members of negative feedback control pathways (*Usp18*, *Irf2*). The activation of a type 1 interferon response by GC infection is a relatively newly described phenomenon and its presence in our transcriptional study highlights its potential importance in the host response to gonococcal infection [52]. In the context of this study, increased activity of type 1 interferons only during diestrus infection suggests that these processes may be, in addition to the previously described differential activation amplitude of the common anti-gonococcal response, responsible for the profound phenotypic differences in GC infection at distinct reproductive cycle phases.

### GC infection in diestrus phase induces greater expression fold changes of molecular components of granulocyte trafficking

**Fig. 4:**
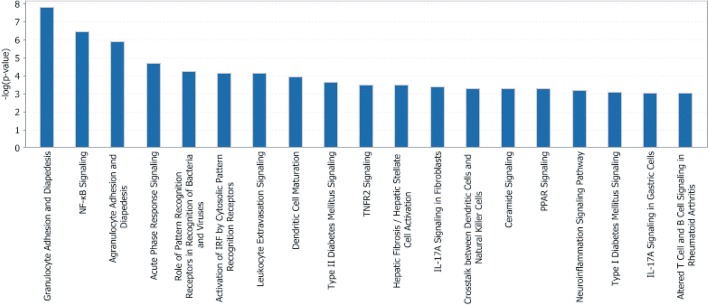
Canonical pathways significantly associated with phase-dependent response to infection. Ingenuity Pathway Analysis was used to evaluate biological pathways that are significantly overrepresented within the 416 genes with phase:infection FDR *q* < 0.25. Canonical pathways with *p* < 0.001 by Fisher’s exact test are shown

**Fig. 5:**
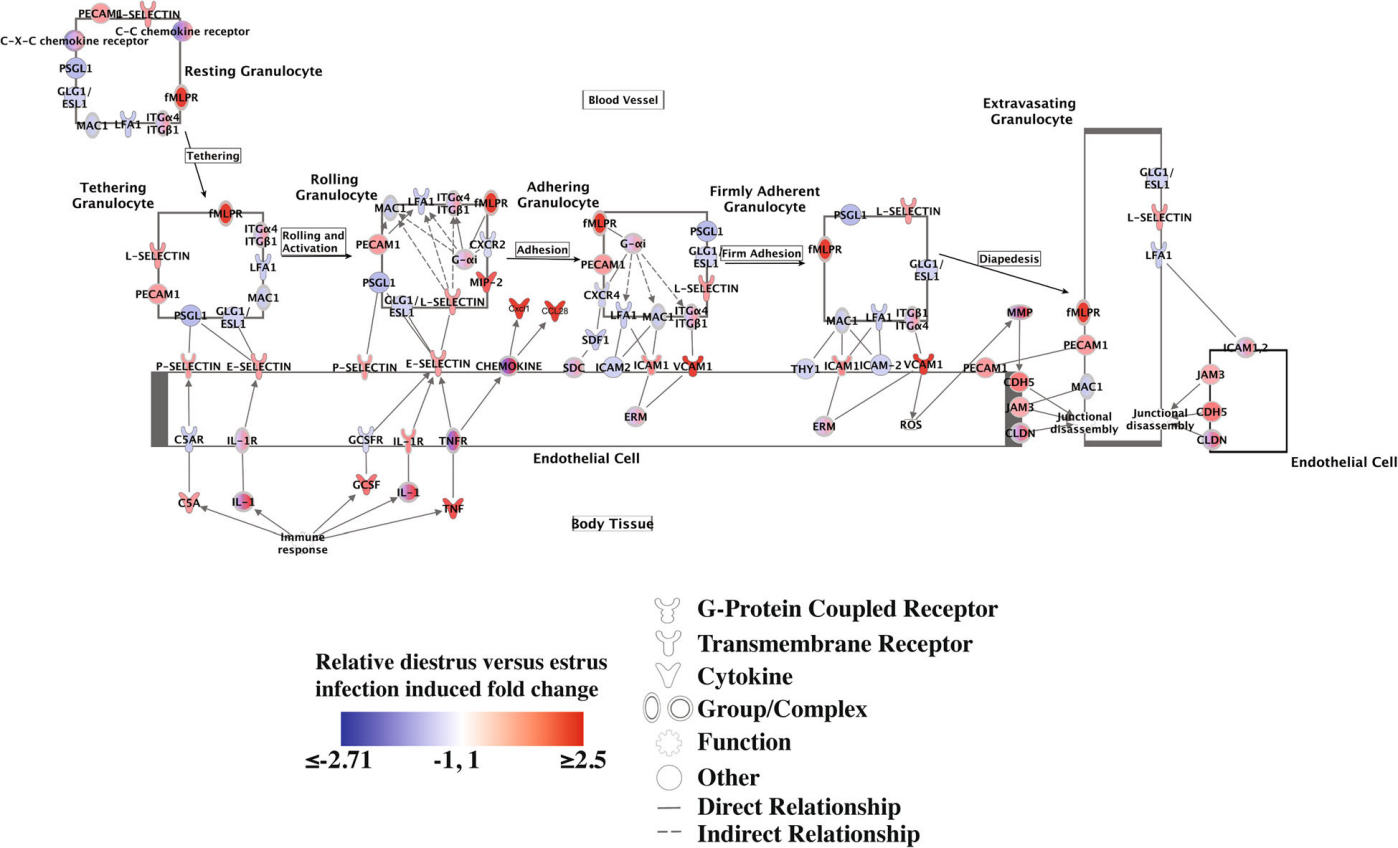
Granulocyte activation and diapedesis mediators show greater induction during diestrus phase infection than in estrus. The members of the Ingenuity Pathway Analysis “Granulocyte Activation and Diapedesis” pathway are shaded according to the differential fold change in transcript level after 6 h of transcervical infection with 10^7^ gonococci during diestrus versus estrus phase over phase specific controls (signed ratio of infection-induced fold change in diestrus phase to that in estrus phase). Colors are scaled so that red and blue indicate differential fold changes of ≥2.5 or ≤ − 2.71, respectively, and white indicates a differential fold change of 0 (no difference in fold change between phases). N = 4 for all conditions except PBS estrus N = 3

We employed Ingenuity Pathway Analysis (IPA) to further evaluate the biological pathways represented in significant (FDR *q* < 0.25) phase:infection interaction-effected genes. IPA identified, among other pathways, adhesion and diapedesis pathways for both granulocytes (neutrophils, basophils and eosinophils) and agranulocytes (lymphocytes and monocytes) as significantly differentially induced in diestrus infection compared to estrus infection (Fig. 4). Since our current transcriptional analysis suggests a differential induction of a chemokine-centric common anti-gonococcal response may be partially responsible for the previously reported differences in granulocyte infiltration of infected tissue between diestrus and estrus phases [33], we chose to examine the granulocyte pathway more closely (Fig. 5).

**Fig. 6:**
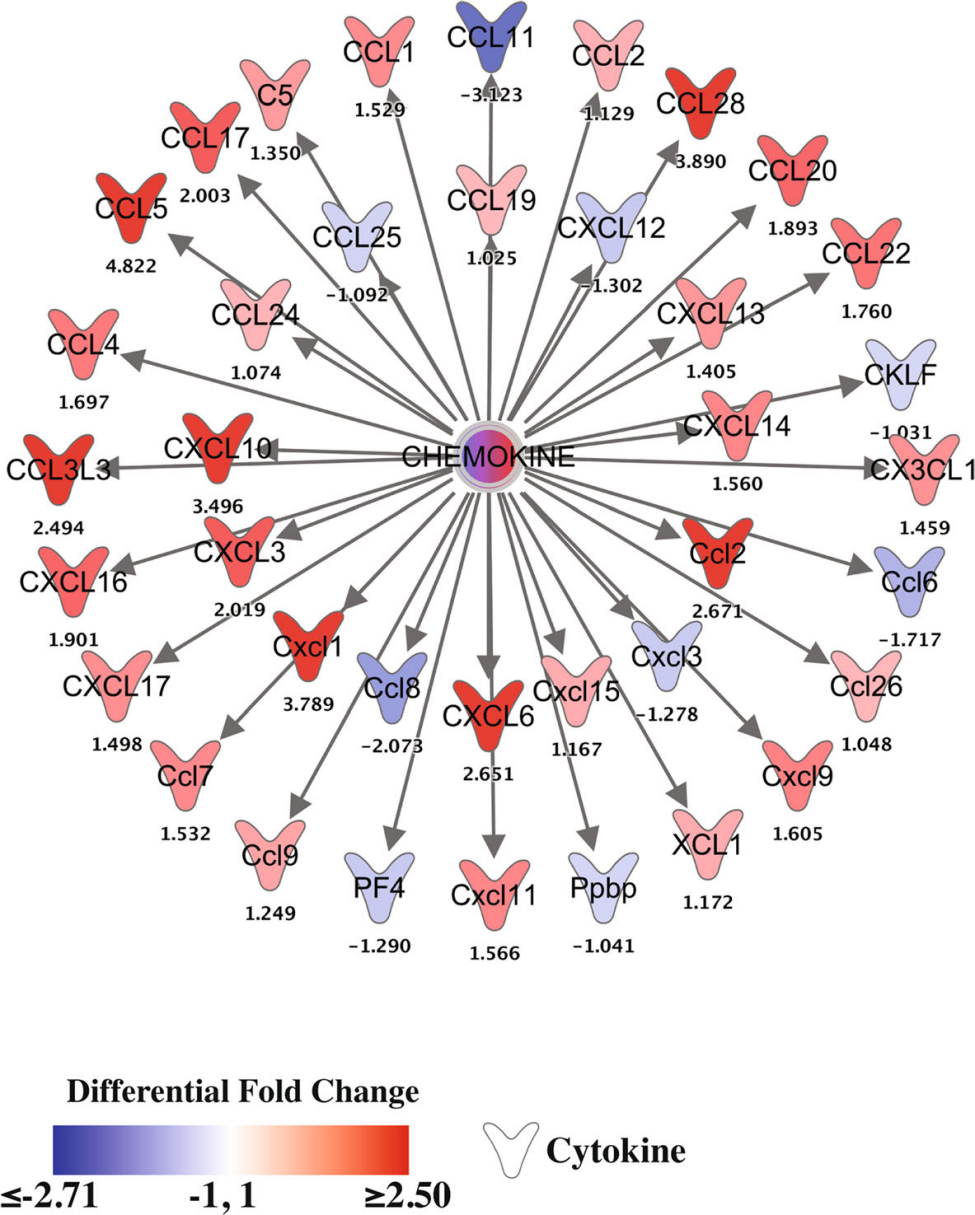
Chemokines generally show greater induction during infection in diestrus phase than in estrus phase. The phase-dependent effect on infection induced chemokine expression is shown as differential fold change in transcript level after 6 h of transcervical infection with 10^7^ gonococci during diestrus versus estrus phase over phase specific controls (signed ratio of infection-induced fold change in diestrus phase to that in estrus phase). Colors are scaled so that red and blue indicate differential fold changes of ≥2.5 or ≤ − 2.71, respectively, and white indicates a differential fold change of 0 (no difference in fold change between phases). N = 4 per condition except for PBS estrus N = 3

The chemokine components of the granulocyte adhesion and diapedesis pathway showed some of the greatest differences in phase-dependent infection induction (Fig. 6), including the primary neutrophil chemokine Cxcl1 and the strong mucosal lymphocyte chemokine Ccl28 [53, 54], which had differential fold change (DFC; i.e., ratio of fold change during diestrus-phase infection to fold change during estrus-phase infection) values of 3.8 and 3.9, respectively. In addition to classic chemokines, the gene Fpr2, which encodes a receptor for the potent neutrophil chemoattractant formyl-methionyl-leucyl-phenylalanine (fMLPR) [55–58], is also upregulated by infection in a phase-dependent manner (DFC of 5.7). Similarly, genes involved in neutrophil rolling, adhesion (the selectins Sell and Selp and the adhesion molecules Icam1, Pecam1, and Vcam1) [59–64] diapedesis and transmigration (e.g., Cdh5 and Jam3) [65, 66] showed greater positive induction in diestrus than estrus infection, generating DFC values ranging from 1.2–2.5. Taken together, these observations describe a highly activated endothelium interacting with a large, chemokine-mobilized population of granulocytes, leading to increased movement of cells into infected tissues. These observations help to clarify elements of the driving mechanism behind the reproductive-cycle-phase-dependent phenotypic differences in host response to GC infection.

## Discussion

For more than two decades, the primary laboratory model of genitourinary *Neisseria gonorrhoeae* (GC) infection has been intravaginal murine infection, which is permissive to colonization only during the estrus phase of the reproductive cycle. While murine vaginal infection with GC during diestrus is unproductive [30, 67, 68], we recently demonstrated [33] that direct transcervical infection of the upper genital tract during this reproductive phase, circumvents the vaginal resistance to GC infection, and elicits an overt uterine pathology including profound inflammation and robust adaptive immune engagement. Interestingly, this inflammatory response is not an obligatory outcome of uterine infection since there is little clinically or histologically-evident response to transcervical infection during estrus. This study extends our previous analyses of uterine cellular composition and cytokine levels during infection [33] by aiming to understand transcriptional differences underlying the strikingly different phenotypic outcomes of acute (6 h post infection) transcervical gonococcal infection during the estrus and diestrus phases.

The induction of cytokine expression, particularly chemokines, by GC infection has been previously described repeatedly [29, 31–33, 56]. This study expands and clarifies the host response to GC through transcriptional analysis, identifying primarily chemokine pathways as significantly induced by GC infection. Due to the nature of our analysis, we were able to evaluate differential expression and therefore biological pathway activation due to the independent effect of just one of the experimental variables, reproductive phase and infection status, or due to a combined phase:infection interaction effect. It was through this analysis that we were able to show that a very similar profile of biological pathways is induced in response to GC infection regardless of during which reproductive phase infection occurs. This observation is significant in light of our previously described profound differences in infection phenotype during diestrus phase and estrus phase [33]. This suggests that, despite the different natural histories of infection that have been described, a chemokine-centric induction of cytokines may be the foundation of a common anti-gonococcal host response. Reflecting this, leading-edge genes from the “Chemokine Receptors Bind Chemokines” pathway, identified by infection effect GSEA (Additional file 3), demonstrate clear phase independent induction (Fig. 1). In addition, several of these same chemokines are found among the leading-edge genes of a top phase:infection interaction effect GSEA gene set, “Chemokine activity” (Fig. 3a). This general anti-GC response, in addition to reflecting the human and mouse data found in the literature, is reminiscent of the clinical picture of gonorrhea as well, characterized by local inflammation and influx of granulocytes into infected tissue.

While induction of these pathways by GC infection is phase independent, the magnitude of induction does not seem to be. The same cytokine and cell recruitment pathways induced in infected estrus tissue, undergo a much greater induction of gene expression in infected diestrus tissue. This can be appreciated in the infection effect GSEA leading edge heatmaps (Fig. 1), with most genes showing an appreciably greater change during diestrus infection compared to uninfected controls. This phase-dependent differential induction of the pathways identified in our infection effect analysis would explain the inclusion of similar pathways in our transcriptional analysis of phase:infection effected genes and pathways (Table 2 and Fig. 3). Additionally, closer examination of the leading-edge genes of significant chemokine gene sets shows significant representation of neutrophil chemokines (Cxcl5, Ccl4 and Cxcl1), suggesting strong phase:infection interaction effect. Supporting this, the phase:infection effect clustering showed that several potent neutrophil chemokines demonstrated a diestrus-specific induction expression pattern (Fig. 2c). This would explain, at least in part, the significant differences in neutrophil recruitment during GC infection in different reproductive cycle phases. A model of granulocyte adhesion and diapedesis was able to confirm, not only greater activation of the pathway during diestrus infection, but that the class of molecules which appeared to most contribute to this difference was chemokines and, unexpected but consistent with this, mediators of diapedesis (Fig. 5).

While differential induction of a chemokine-centric common anti-gonococcal host response appears to explain a portion of the described differences in murine transcervical GC infection during estrus and diestrus reproductive phases, linear modeling of phase:infection interaction effect suggests additional pathways may contribute. In contrast to the induction of common chemokine activities during GC infection in both phases, the induction of interferon pathways appears to be largely unique to GC infection in diestrus phase. Although the activation of these pathways during GC infection has been reported before [52, 69], it is a phenomenon that is much less understood than the previously discussed cytokine response. Those studies that have examined the impact of type 1 interferons in GC infection have suggested, based on impaired bacterial killing in the context of IFN-β, that it has a detrimental effect on infection control and resolution [52]. A negative impact of type 1 interferon on an antibacterial response has been described for several other human pathogens, including the genito-urinary pathogen *Chlamydia trachomatis* [70]. Despite this, the role of type 1 interferons may not be quite that simple. Another member of the type 1 interferon family, IFN-ε, has also been shown to be induced in GC infection and is a known mediator of female genital tract immunity [71]. Although the role of type 1 interferon signaling in GC infection is unclear and likely complex, the significant and specific induction of related pathways in diestrus phase infection, where greater pathology is observed, suggests that they might contribute a detrimental effect on the host. The emergence of these immune processes in our transcriptional analysis reinforces their potential importance and supports the further exploration of the role of type 1 interferons in human gonorrhea and the murine model of disease.

## Conclusions

In this study, we present evidence to suggest that the reproductive cycle has a profound effect on the transcriptomic response to uterine infection with *Neisseria gonorrhoeae*. Although a chemokine focused induction of cytokine expression and function was observed in all infected tissue, the magnitude of this common anti-gonococcal response was phase-dependent. There was significantly greater expression of immune cell recruitment molecules, particularly those that target neutrophils, when infection occurred in the diestrus phase. In fact, the granulocyte adhesion and diapedesis biological pathway demonstrated general greater activation in diestrus phase infection as compared to estrus phase infection. This differential response likely drives the greater tissue infiltration of neutrophils during diestrus infiltration that we described in our previous work. While neutrophil infiltration was perhaps the most striking difference between infection in diestrus and estrus phase, there also appeared to be greater inflammation and loss of mucosal integrity during diestrus. Our work here cannot fully explain the molecular cause of these additional differences, but we were able to identify a diestrus infection specific activation of type 1 interferon pathways that have been implicated as host-detrimental in some anti-bacterial responses; this raises the question of its effect in *N. gonorrhoeae* infection. These findings help clarify the underlying biological processes that characterize the anti-gonococcal response, both protective and potentially destructive, in the murine transcervical *N. gonorrhoeae* infection model, thereby providing new avenues to evaluate the efficacy of next generation GC treatments and vaccines as well as future studies of the natural infection process.

## Methods

### Mouse strains

Mice were 6-week-old female wild-type FVB animals purchased from Charles River (Canada). Mice were allowed to acclimate for 1–2 weeks following arrival before entering the experimental protocol.

### Reproductive cycle staging

Starting 5 days prior to infection, each mouse was evaluated daily for reproductive cycle phase by cytological analysis of wet mounts [72]. Slides were prepared from 30 μl phosphate-buffered saline (PBS, Life Technologies, Burlington, Canada) vaginal washes and viewed under a 40× objective.

### Bacterial strains

*Neisseria gonorrhoeae* used for infection experiments were low passage isolates originally collected during a longitudinal study of commercial sex worker in Nairobi, Kenya [33, 73]. Bacteria was grown on GC agar (Becton Dickinson, Sparks, USA) supplemented with IsoVitalex (Becton Dickinson, Sparks, USA) at 37 °C in a humidified 5% CO_2_ atmosphere.

### Murine transcervical infection

A single strain culture of *Neisseria gonorrhoeae* was grown overnight on a chocolate agar plate to produce a lawn of bacterial colonies. A full plate was collected into 1 mL of PBS supplemented with 0.9 mM CaCl_2_ and 0.5 mM MgCl_2_ (PBS^++^, Life Technologies, Burlington, Canada). The OD_550_ absorbance of the bacterial suspension was measured and used to calculate the concentration of gonococci. This initial suspension was then diluted with PBS^++^ to produce a 5 × 10^8^ gonococci per milliliter suspension. Mice of known reproductive cycle phase were then anesthetized via inhalation of isoflurane. Infection was achieved as previously described [33]. Briefly, anesthetized mice were laid prone at a 45-degree angle and, using a blunted 25-gauge needle, 20 μl of the infection suspension (10^7^ gonococci) was delivered directly into the uterine horns. Six hours after infection, the mice were sacrificed by CO_2_ asphyxiation. Sera were obtained via cardiac puncture. Lower and upper genital tract tissues were removed and separated at the point where the cervix joins the uterine body. Collected tissue was frozen using liquid nitrogen and stored at -80 °C until analyzed.

### Tissue processing

Frozen tissue samples were thawed and divided evenly for protein or RNA extraction. Tissue processed for RNA was placed in TRIzol and homogenized using QIAshredder tissue homogenizer kits (Qiagen Cat#79654). RNA was extracted from the tissue homogenate using a RNeasy Mini kit (Qiagen Cat# 74104). Isolated nucleic acid was initially analyzed for purity and integrity by 280/260 absorbance ratio via Nanodrop. Samples were then frozen at -80 °C until ready for use.

### Microarray

RNA expression was profiled by the Boston University Microarray and Sequencing Resource using Affymetrix Mouse Gene 2.0 ST microarrays. Samples were processed in two batches of nearly identical size and representation of experimental groups to reduce any batch effect. Biotin labeling was performed using the WT Plus reagent kit (Affymetrix, Santa Clara, CA) according to the manufacturer’s protocol. The labeled, fragmented DNA was hybridized to the Affymetrix Mouse Gene 2.0 ST Array for 18 h in a GeneChip Hybridization oven 640 at 45 °C with rotation (60 rpm). The hybridized samples were washed and stained using an Affymetrix fluidics station 450. After staining, microarrays were immediately scanned using an Affymetrix GeneArray Scanner 3000 7G Plus. Raw and processed microarray data have been deposited in the Gene Expression Omnibus (GEO), Series GSE113962.

### Quality assessment

Prior to analysis of expressional data, the quality of the microarrays was assessed using two metrics: Relative Log Expression (RLE), which indicates whether the distribution of intensity values of a relatively dim array have been artificially skewed upwards by the Robust Multiarray Average (RMA) normalization algorithm, and Normalized Unscaled Standard Error (NUSE), which is a measure of the noise inherent in the estimate of each probeset (gene). The median RLE values were relatively similar across 13 of the samples (range − 0.05 to 0.05), as were the median NUSE values (range 0.99 to 1.02). However, the remaining two samples (GC-infected estrus phase samples 6 and 7) had higher median RLE (0.092 and 0.105, respectively) and NUSE (1.03 and 1.04, respectively) values, indicating that these two arrays may be of lower quality compared to the rest of the experiment.

### Assessment of and correction for array batch effect

**Fig. 7:**
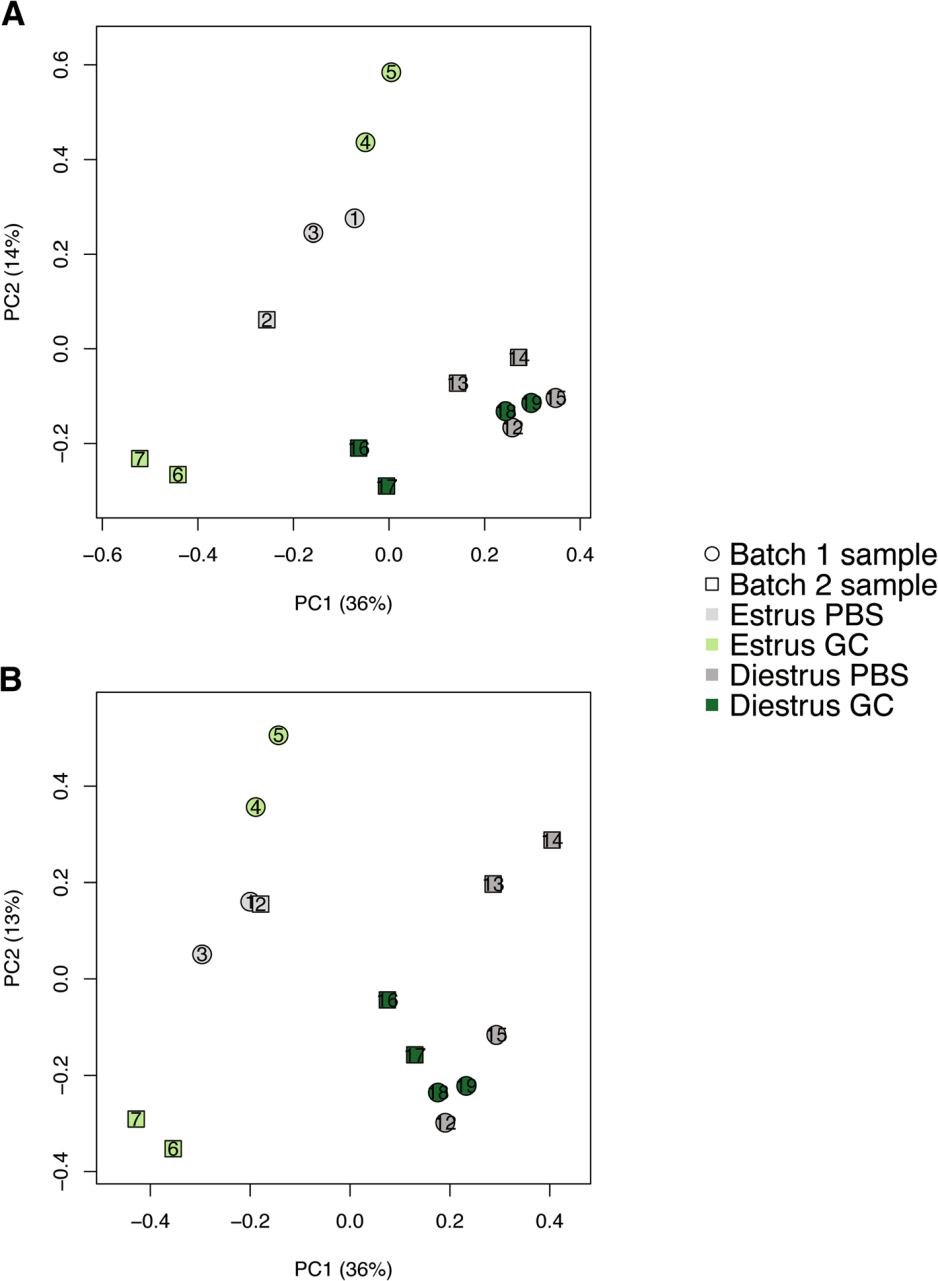
Principal Component Analysis (PCA) before and after batch correction. All samples are plotted with respect to the first and second Principal Components (PC), computed using log2 (expression) values *z*-normalized across all samples (to a mean of zero and a standard deviation of one). PCA was performed both prior to (**a**) and following (**b**) correction for array batch effect using ComBat. Light and dark colors indicate estrus-phase and diestrus-phase samples, respectively, and gray and green indicate PBS-treated and GC-infected samples, respectively. Samples from array batches 1 and 2 are plotted as circles and squares, respectively. N = 4 for all groups except PBS Estrus N = 3

Because the arrays were processed in two separate batches, Principal Component Analysis (PCA) was employed to assess the strength of batch effect (Fig. 7a). The samples cluster primarily by reproductive-cycle phase, but separate within each phase primarily by batch, indicating that a substantial batch effect is initially present. In order to correct for this effect, expression values were adjusted using the ComBat algorithm, and PCA was repeated (Fig. 7b). Following batch adjustment, the samples again separate well by reproductive-cycle phase, but within the diestrus phase group, greater separation by treatment was seen. The GC-infected estrus samples 6 and 7, which had been identified as being of lower quality, still separated from GC-infected estrus samples 4 and 5 along the PC2 axis, indicating that batch adjustment did not fully account for the relative difference in quality between these two pairs of samples. Despite persistence of moderate batch effect, samples 6 and 7 were retained for analysis, since their median RLE and NUSE values were not drastically higher than the rest of the arrays, and without them, batch one GC-infected estrus arrays would be unopposed by any batch two arrays. The potential loss of array sensitivity due to remaining batch effect, is outweighed by the larger effect of an unopposed batch effect. In addition, the phenotypic differences described in this model suggests that there are major differences in induction of biological pathways that may still be identified even in a slightly less sensitive system. Indeed, significant transcriptional differences were identified indicating a non-critical impact by the residual batch effect following ComBat adjustment.

### Microarray analysis

Mouse Gene 2.0 ST CEL files were normalized to produce gene-level expression values using the implementation of the Robust Multiarray Average (RMA) [74] in the affy package (version 1.36.1) [75] included in the Bioconductor software suite (version 2.12) [76], and an Entrez Gene-specific probeset mapping (17.0.0) from the Molecular and Behavioral Neuroscience Institute (Brainarray) at the University of Michigan [77]. Array quality was assessed by computing Relative Log Expression (RLE) and Normalized Unscaled Standard Error (NUSE) using the affyPLM package (version 1.34.0). The implementation of the ComBat algorithm in the sva package (version 3.4.0) was used to adjust the expression values for the batch in which the arrays were scanned, adjusting for phase, infection, and the interaction of the two (phase:infection) as covariates. Differential expression was assessed using the moderated (empirical Bayesian) t test implemented in the limma package (version 3.14.4) (i.e., creating simple linear models with lmFit, followed by empirical Bayesian adjustment with eBayes). Correction for multiple hypothesis testing was accomplished using the Benjamini-Hochberg false discovery rate (FDR). Human homologs of mouse genes were identified using HomoloGene (version 68) [78]. All microarray analyses were performed using the R environment for statistical computing (version 2.15.1).

### Gene set enrichment analysis

GSEA (version 2.2.1) [35] was used to identify biological terms, pathways and processes that are coordinately up- or down-regulated within each pairwise comparison. The Entrez Gene identifiers of the human homologs of the genes interrogated by the array were ranked by the *t* statistic computed between Ng and PBS within each reproductive-cycle phase, or by the treatment or phase:treatment *t* statistic. Mouse genes with multiple human homologs (or vice versa) were removed prior to ranking, so that the ranked list represents only those human genes that match exactly one mouse gene. This ranked list was then used to perform pre-ranked GSEA analyses (default parameters with random seed 1234) using the Entrez Gene versions of the Hallmark, Biocarta, KEGG, Reactome, Gene Ontology (GO), and transcription factor and microRNA motif gene sets obtained from the Molecular Signatures Database (MSigDB), version 5.0 [34].

### Ingenuity pathway analysis (IPA)

Canonical pathways were automatically identified using the 416 genes with FDR *q* < 0.25 for the phase:infection interaction *t* test (computed after removing genes that were not expressed above the median value of at least one array). Analysis was performed using IPA’s reference database and all Ingenuity-supported third-party databases, set to consider direct molecular relationships, allowing for experimentally observed and predicted relationships with high confidence, and restricted to mouse tissue and cell lines. Differential fold changes were calculated by computing fold changes (infected versus uninfected) within each reproductive cycle phase and then obtaining the ratio of the two (diestrus:estrus). The granulocyte adhesion and diapedesis pathway figure was built using IPA Path Designer with differential infection-induced fold change data overlaid.

## Additional files

Additional file 1: This file contains the full, analyzed data set including results of all moderated t-tests as well as raw log2 expression values for all gene probes. (XLSX 22480 kb)

Additional file 2: File contains full results from GSEA analysis of phase effect genes in table form. (XLSX 282 kb)

Additional file 3: File contains full results from GSEA analysis of treatment effect genes in table form. (XLSX 281 kb)

Additional file 4: File contains full results from GSEA analysis of phase:treatment interaction effect genes in table form. (XLSX 286 kb)

## Abbreviations

CDC: Centers for disease control and prevention
DFC: Differential Fold Change
FDR: False discovery rate
GC: Gonococcal/*Neisseria gonorrhoeae*
GEO: Gene Expression Omnibus
GSEA: Gene Set Enrichment Analysis
HIV: Human immunodeficiency virus
IPA: Ingenuity Pathway Analysis
MIC: Minimum inhibitory concentration
MSigDB: Molecular Signatures Database
NIH: National Institutes of Health
NUSE: Normalized Unscaled Standard Error
PCA: Principal component analysis
PID: Pelvic inflammatory disease
PRR: Pattern recognition receptor
RLE: Relative log expression
RMA: Robust Multiarray Average
UGT: Upper genital tract
WHO: World Health Organization
